# Congenital glaucoma: the ‘not-so-silent’ thief of sight in children

**Published:** 2024-02-09

**Authors:** Vera Adobea Essuman, Vera Mawusime Beyuo

**Affiliations:** 1 Professor of Paediatric Ophthalmology: University of Ghana Medical School, Accra, Ghana.; 2 Paediatric Ophthalmologist: Lions International Eye Centre, Korle Bu Teaching Hospital, Accra, Ghana.

**Congenital glaucoma is an important cause of blindness in very young children, but vision can be preserved if it is identified and treated as early as possible.**

Although uncommon, congenital glaucoma is an important cause of blindness in children, particularly in low-resource settings, where children are often only seen once the disease has progressed and parents find it difficult to attend regular, long-term follow-up visits. Early detection and treatment, which is usually surgical in the first instance, can preserve vision.

## What is congenital glaucoma?

**Figure 1:**
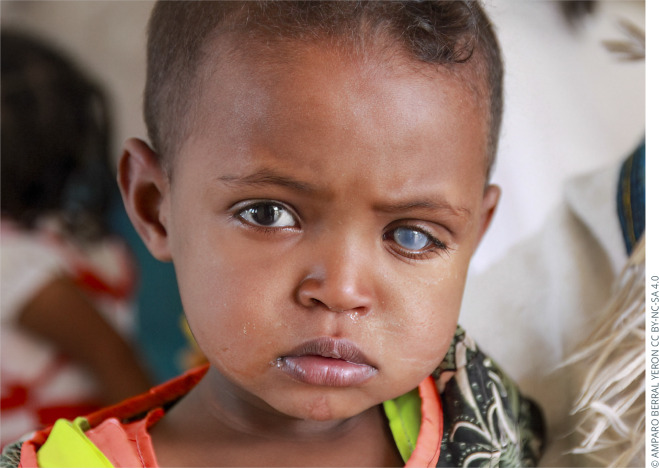
A child who presented late with congenital glaucoma in the left eye. The whole eye, including the cornea, is enlarged (bupthalmos) and the cornea is hazy. ethiopia

**Figure 2:**
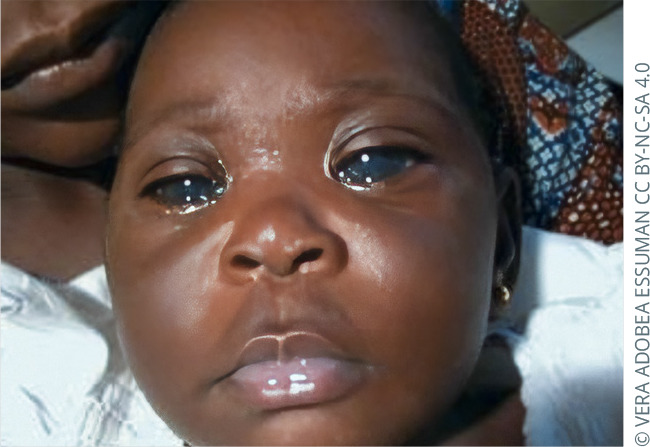
A baby with enlarged eyes (buphthalmos), enlarged, hazy corneas, and excessive watering (epiphora) in both eyes. ghana

Congenital glaucoma, which is usually bilateral, can be present at birth or become apparent during the first few years of life. In congenital glaucoma, high intraocular pressure (IOP) causes the eye(s) to enlarge (unlike in adults or older children where the size of the eye does not change). This enlargement of the eye is known as buphthalmos, or ‘ox eye’ (Figures 1 and 2). Congenital glaucoma causes progressive damage to the optic nerves and cornea, as well as thinning of the sclera. The incidence varies with ethnicity, ranging from 1 in 2,500–8,200 live births in the Middle East to 1 in 10–20,000 live births in Western populations.^1^,^2^ Worldwide, congenital glaucoma is responsible for 5–18% of blindness in children.^3^

## Definitions and causes

Congenital glaucoma can be primary or secondary, according to the 9th World Glaucoma Association Consensus.^4^

### Primary congenital glaucoma

This is caused by genetic mutations, leading to abnormal development of the trabecular meshwork, resulting in obstruction to aqueous outflow and high IOP. The genes involved include CYP1B1 and LTBP2.^4^ Consanguinity (marriage between close relatives such as cousins) increases the risk.

In some children, the condition may be apparent from birth. In other children, the eyes may appear normal at birth and the condition only becomes apparent later. Subcategories of primary congenital glaucoma are therefore based on the age of onset:

- Neonatal onset (birth to 1 month)
- Infantile onset (>1 month to 24 months)
- Late onset or late recognised (>2 years).

If children have the typical signs of primary congenital glaucoma, but these do not progress, and they have normal IOP and optic discs, their condition is classified as spontaneously arrested congenital glaucoma.

### Secondary congenital glaucoma

Secondary congenital glaucoma is associated with a variety of ocular and systemic syndromes, mainly:

- Other congenital eye anomalies, such as Peter's anomaly or aniridia, which may or may not be associated with systemic anomalies
- Systemic diseases or syndromes, such as Sturge-Weber syndrome, neurofibromatosis, and congenital rubella syndrome, which may be associated with eye signs, including signs of congenital glaucoma.

It is important to distinguish between primary and secondary congenital glaucoma. In secondary congenital glaucoma, the IOP can be harder to control, and the outcomes of management can be worse. Affected children also need to be referred to a paediatrician to manage any systemic abnormalities, such as heart defects associated with congenital rubella syndrome.

## What to look for at the local/primary level and when to refer

Mothers typically say that they have noticed three things wrong with their child's eyes:

1. Excessive watering (epiphoria, see Figure 2)
2. Avoidance of bright light (photophobia)
3. Excessive blinking (blepharospasm).^4^

They may also notice that the corneas look hazy.

Signs to look for include:

- Enlargement of the eye(s), known as buphthalmos
- Enlargement of the cornea
- Corneal haziness due to oedema.

The child may also show signs of distress due to pain.

In most low-resource settings, children present late, with signs of advanced disease with marked corneal haziness or opacity. **Any children in whom glaucoma is suspected should be referred urgently to an eye department or hospital where there is a paediatric ophthalmologist.**

## Diagnosis at the tertiary level

At the tertiary (or teaching hospital) level, it is important to make the diagnosis and identify whether the child has primary congenital glaucoma or secondary congenital glaucoma. This is because the management can be different, and the prognosis is usually better for primary congenital glaucoma. A multidisciplinary team is needed, including anaesthetists, paediatricians, orthoptists, optometrists, counsellors, and possibly social workers.

The diagnosis is made clinically.

- Ask the parents or carers about other problems which may help to distinguish between primary or secondary congenital glaucoma. For example, whether the parents are close relatives (suggestive of primary glaucoma), whether the baby had a rash at birth (suggestive of congenital rubella, a cause of secondary glaucoma), or whether the parents have noticed any other problems from birth (also suggestive of secondary glaucoma).
- Examination should include a systemic examination, looking for signs which suggest that the child may have secondary congenital glaucoma.
- Measure the visual acuity, if possible, and examine the anterior and posterior segments of the eyes.
- Sedation or anaesthesia is helpful to enable a thorough examination. Oral chloral hydrate, 50–80 mg/kg, can be used for sedation, with the advantage that it does not lower the IOP very much.
- The anterior segment can be examined with a hand-held slit lamp or torch and a 15D or 20D lens. Look for splits in Descemet's membrane in the cornea (Haab's striae), which indicate that the cornea has enlarged as a result of high IOP. Note any thinning of the sclera and whether a staphyloma is present.
- To assess whether the eye or eyes are larger than normal, measure the horizontal corneal diameter, from ‘white to white’ limbus, using callipers. The cornea is enlarged if it is wider than the values below:
  - >11 mm in a newborn
  - >12 mm in infant aged 1 to 12 months
  - >13 mm (any age)
- Note any corneal haze. Grade this if possible, as grading helps with follow-up assessment and may be important for medicolegal reasons.
- Measure IOP using a Perkins tonometer, a Tonopen, or an iCare device.
- Posterior segment examination is possible if the cornea is clear enough to visualise the optic disc. In case there is poor visualisation due to corneal oedema, the view of the posterior segment can be improved by gently removing the corneal epithelium if the child is being examined under general anaesthesia, prior to gonioscopy. In congenital glaucoma, optic disc cupping tends to be circumferential initially, as it appears the cupping is due to stretching of the sclera posteriorly. However, there may be areas of focal thinning and atrophy as the disease progresses.
- The Congenital Glaucoma Research Network (CGRN)^4^ recommends that gonioscopy should be done at least once. This helps to identify changes, sometimes very subtle, that are suggestive of a secondary cause.

## Other investigations at tertiary level

The following investigations are useful to support the diagnosis and to monitor the effectiveness of treatment.

**Refraction.** Hyperopia that is below the expected range for their age, or myopia in an infant in the presence of other signs, can confirm the diagnosis of congenital glaucoma. A change in the refractive error over time, particularly if the refraction becomes more myopic, implies that the eyes are getting longer as a result of inadequate IOP control.

**“Any children in whom glaucoma is suspected should be referred urgently to an eye department or hospital where there is a paediatric ophthalmologist.”**

**Ultrasound to determine axial length.** Axial length measurement using an A-scan is also useful in confirming the diagnosis and monitoring progression. Normal axial length increases from 16.2 mm at term (37 weeks’ gestation) to an average of 21.8 mm at age 3. The rate of growth slows down from 0.62 mm/month (birth to 6 months) to 0.19 mm/month (6–18 months of age), finally slowing to 0.1 mm/month from the age of 18 months onwards.^5^

B-scan ultrasound may reveal intraocular pathology, such as retinoblastoma, responsible for secondary glaucoma. In patients where the discs cannot be visualised due to corneal haze, it can also show whether optic disc cupping is present.

Refraction and axial length measurements can serve as proxies for IOP, as the latter may be difficult to measure at each follow-up visit, particularly in low-resource settings. It is therefore important to gather baseline values by measuring visual acuity and performing an A-scan at the first visit.

## Management at the tertiary level

Angle surgery is the treatment of choice for congenital glaucoma. This includes goniotomy (ab interno approach) or trabeculotomy using ab externo approach (superior, inferior or 360°). Trabeculectomy is usually undertaken if goniotomy fails. In centres where children often present late, combined trabeculotomy and trabeculectomy is the treatment of choice to optimise outcomes. Topical antiglaucoma medications can also be used as a temporary measure before surgery or when additional treatment is needed to control the IOP after surgery. Trabeculectomy can be very challenging in eyes with buphthalmos, as the sclera can be very thin and the anatomy distorted. Only ophthalmologists who have been trained and have experience should perform these operations.

### Follow-up and long-term management

After surgery, the frequency of follow-up is determined by the findings. Repeat examination under anaesthesia or sedation may be needed to monitor IOP, corneal clarity and diameter, axial length, and refraction. It is important to fully correct any refractive error to prevent amblyopia which may lead to poor visual outcomes, even if the IOP is controlled and the corneas have cleared. If amblyopia is suspected, start occlusion therapy as soon as possible.

Counselling parents or caregivers is of vital importance so that they understand the need for regular follow-up, compliance with topical medication if applicable, and that follow-up needs to be lifelong. When the children are older, they should also be counselled.

**“Counselling parents or caregivers is of vital importance so that they understand the need for regular follow-up.”**

### Prognosis and complications

Vision can be preserved with good IOP control as this prevents disease progression and helps protect the optic nerve. Young children with primary glaucoma who present early have the best prognosis. Loss of vision from congenital glaucoma can be due to optic nerve damage, amblyopia, high myopia and its complications, astigmatism, and/or lens dislocation.

**Figure 3:**
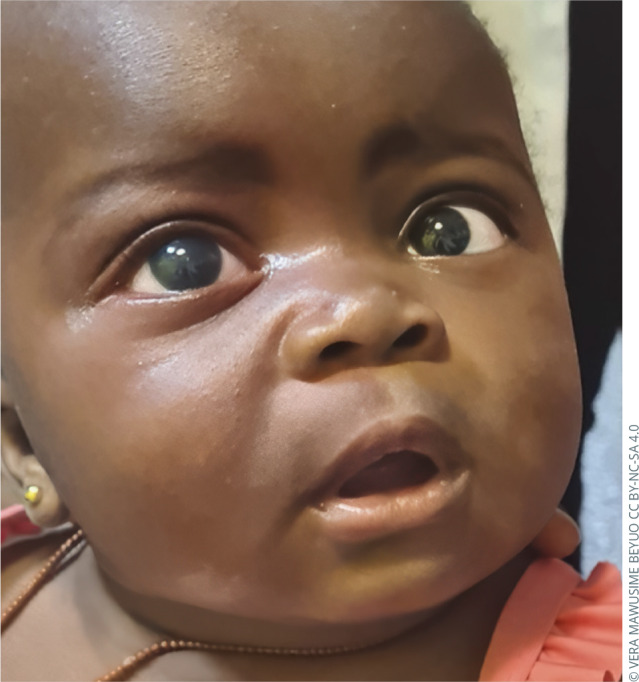
A child with secondary congenital glaucoma in the right eye associated with Sturge-Weber syndrome. Note the following: right hemifacial hypertrophy, port-wine birthmark (difficult to see in dark-skinned children), conjunctival injection, and buphthalmos with an enlarged, hazy cornea. ghana

**Case study**

An 8-week-old infant with neonatal onset congenital glaucoma (Figure 4a) underwent combined trabeculotomy and trabeculectomy operations at 8 weeks in her right eye and at 10 weeks in her left eye. Two years after surgery, she had normal intraocular pressure in both eyes, with relatively clear corneas (Figure 4b). Visual acuity in each eye was 6/12 unaided, and 6/9 with spectacle correction.

**Figure 4a:**
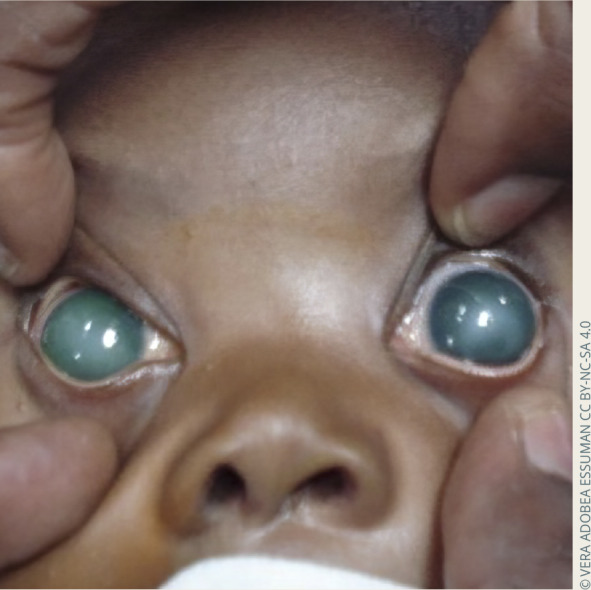
Eight-week-old infant with primary congenital glaucoma (neonatal onset) before surgery. ghana

**Figure 4b:**
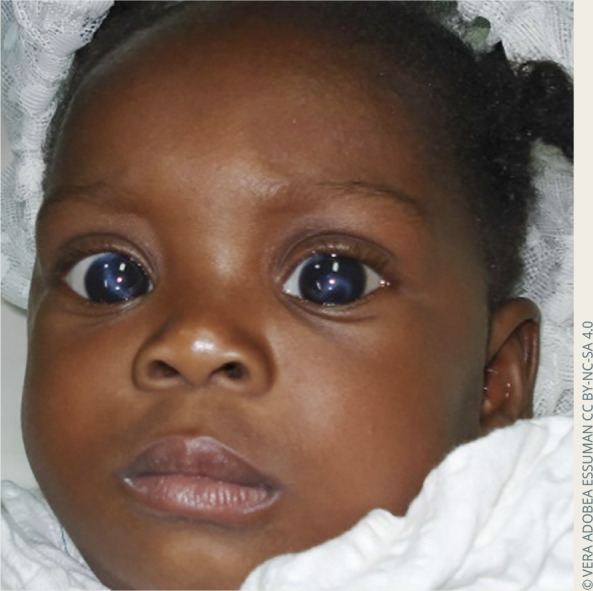
The same child two years after surgery, with controlled disease. ghana
